# Expression of human papilloma virus type 16 E5 protein in amelanotic melanoma cells regulates endo-cellular pH and restores tyrosinase activity

**DOI:** 10.1186/1756-9966-28-4

**Published:** 2009-01-08

**Authors:** Fabio Di Domenico, Cesira Foppoli, Carla Blarzino, Marzia Perluigi, Francesca Paolini, Salvatrice Morici, Raffaella Coccia, Chiara Cini, Federico De Marco

**Affiliations:** 1 Department of Biochemical Sciences, University of Rome "La Sapienza", P.le Aldo Moro, 5 – 00185 Rome, Italy; 2 CNR Institute of Molecular Biology and Pathology, P.le Aldo Moro, 5 – 00185 Rome, Italy; 3 Laboratory of Virology, "Regina Elena Institute for Cancer Research", Via delle Messi d'oro 156- 00156 Rome, Italy

## Abstract

**Background:**

Melanin synthesis, the elective trait of melanocytes, is regulated by tyrosinase activity. In tyrosinase-positive amelanotic melanomas this rate limiting enzyme is inactive because of acidic endo-melanosomal pH. The E5 oncogene of the Human Papillomavirus Type 16 is a small transmembrane protein with a weak transforming activity and a role during the early steps of viral infections. E5 has been shown to interact with 16 kDa subunit C of the trans-membrane Vacuolar ATPase proton pump ultimately resulting in its functional suppressions. However, the cellular effects of such an interaction are still under debate. With this work we intended to explore whether the HPV16 E5 oncoprotein does indeed interact with the vacuolar ATPase proton pump once expressed in intact human cells and whether this interaction has functional consequences on cell metabolism and phenotype.

**Methods:**

The expression of the HPV16-E5 oncoproteins was induced in two Tyrosinase-positive amelanotic melanomas (the cell lines FRM and M14) by a retroviral expression construct. Modulation of the intracellular pH was measured with Acridine orange and fluorescence microscopy. Expression of tyrosinase and its activity was followed by RT-PCR, Western Blot and enzyme assay. The anchorage-independence growth and the metabolic activity of E5 expressing cells were also monitored.

**Results:**

We provide evidence that in the E5 expressing cells interaction between E5 and V-ATPase determines an increase of endo-cellular pH. The cellular alkalinisation in turn leads to the post-translational activation of tyrosinase, melanin synthesis and phenotype modulation. These effects are associated with an increased activation of tyrosine analogue anti-blastic drugs.

**Conclusion:**

Once expressed within intact human cells the HPV16-E5 oncoprotein does actually interact with the vacuolar V-ATPase proton pump and this interaction induces a number of functional effects. In amelanotic melanomas these effects can modulate the cell phenotype and can induce a higher sensitivity to tyrosine related anti-blastic drugs.

## Background

Human Papillomavirus type 16 (HPV-16) is a member of species 9 of the mucosotropic α Papillomavirus genus. Together with a further fifteen α Papillomavirus types, HPV16 is comprised within the so called High Risk anogenital HPV (HR-HPV), that are causally involved in the development of malignant tumors [1]. In particular, HPV 16 is the major etiological agent for cervical cancer[2] and it has also been implicated as a causative agent in a number of carcinomas originating from a variety of other anatomical sites. The oncogenic potentials of HR-HPV types depend on the activity of three transforming genes: E5, E6, and E7. The E6 and E7 proteins are unanimously recognized as the major responsible for virus carcinogenicity [3-5]. Conversely, E5 has been found to have only weak transforming properties and accessory functions [6-8] although indirect evidences point to E5 as an hallmark of HR-HPVs carcinogenicity [9,10].

HPV-16 E5 is a highly hydrophobic membrane protein, 83 amino acids long, located mainly at the Endoplasmic Reticulum (ER) and to a lesser extent on the Golgi apparatus, the plasma membranes and early endosomes [11]. Its expression induces several cellular changes, including enhanced growth factor signalling [12], the activation of mitogen-activated protein kinase pathways [13], anchorage independent growth in immortalized fibroblasts [14], down regulation of MHC Class I and Class II molecules [15,16]. Despite the above wide range of activities and in contrast to E5 of Bovine Papillomavirus 1 – one of the first PV oncoproteins to be identified and known as the main oncogene – the biological activities of the HPV16 E5 protein still remain poorly characterized and its role in HPV pathogenesis is far to be understood [17]

While biochemical interaction of the E5 oncoprotein with the vacuolar H^+^-ATPase (V-ATPase) is well accepted the cellular effects of this interaction are still under debate. The V-ATPase, the universal proton pump of eukaryotes, is a major modulator of endoplasmic and endosomal pH and through this modulation it regulates the organellar trafficking and functions. It is known that the E5 protein of HPV-16 can interact with the 16 kDa subunit C of the proton pump [11-18] within the ER where most of the E5 is found. Such an interaction prevents the Subunit C from participating in the assembly of the Vacuolar Subcomplex (V_0 _Subcomplex) that is required for the formation of the mature V-ATPase on the vacuolar membranes [19]. This significantly delays the proteolytic endosomal degradation of the internalized EGFr that eventually recycles to the plasma membrane. This extend the EGFr lifespan and increases the EGF dependent/EGFr signalling [20,21] suggesting that the interaction with the subunit C represent an elective function of E5. Conversely, other authors believe that the impairment of V-ATPase and consequent delayed degradation of internalized EGFr is an indirect result of trafficking disruption and impaired fusion of early endosomes with late acidic endosomes [22,23]. The pH modulation is very important in the regulation of cell organellar trafficking and function in many cellular strains. In particular intra-melanosomal pH has been indicated as an essential factor for the control of melanin deposition in melanocytes [24]. Melanogenesis is regulated through the modulation of tyrosinase, the rate-limiting enzyme of the melanogenic pathway. Differences in tyrosinase activity of melanocytes from different skin photo types (Caucasian or Black skin) have been reported [25]. It has also been shown that these differences were not due to variations in tyrosinase abundance or gene activity, but to the regulation of catalytic activity of the enzyme [25]. In fact, near neutral melanosomal pH is optimal for human tyrosinase activity and melanogenesis while melanin production is suppressed in Caucasian melanocytes by low melanosomal pH [24]. Accordingly, tyrosinase mRNA and tyrosinase protein are actually present also in amelanotic melanomas, where no tyrosinase activity and no melanin deposition can be detected [26,27]. The probable reason of the declined catalytic activity in these cells, where tyrosinase is present in a inactive state, is the low internal pH due to elevated V-ATPase activity consequent to elevated glycolysis and extra-cellular acidification occurring during the metastatic spread. Accordingly, it has been demonstrated that substances that act as selective inhibitors of V-ATPase [28,29] are able to determine the re-activation of tyrosinase and melanogenesis and melanotic reversion of amelanotic melanomas [26].

In the present work we expressed the HPV 16 E5 protein in two lines of human, tyrosinase-positive, amelanotic melanomas with the aim to examine whether the E5 expression could modulate the melanosomal pH and tyrosinase activity. Here we provide evidence that HPV-16 E5 protein inhibits proton pump, causing alkalinisation of endocellular pH, tyrosinase activation, melanin deposition and modulation of sensitivity to dopamine mimetic drugs.

## Methods

### Materials

Concanamycin A (ConA), 3- [4,5-dimethylthiazol-2-yl]-2,5-diphenyl tetrazolium bromide (MTT), 3,4-dihydroxybenzylamine (DHBA) and buthionine sulfoximine (BSO) were purchased from Sigma Chemical Co. (St. Louis, MO, USA). [^3^H] tyrosine was purchased from Amersham Biosciences Ltd, Amersham UK). Dulbecco's modified Eagle's medium (DMEM), RPMI 1640 medium and foetal bovine serum (FBS) were purchased from Invitrogen SRL (San Giuliano Milanese, Italy), as well as the SuperScript One-Step RT-PCR System with Platinum Taq DNA Polymerase. The LZRSpBMNZ and the LZRSpBMNZ-E5 plasmids were kindly provided by G. Sibbett (The Beatson Institute for Cancer Research, Glasgow, UK) [30]. All other reagents were analytical grade products.

### Cell cultures

Two established cell lines of human melanoma, kindly provided by Dr. G. Zupi (Laboratory of Chemotherapy, Regina Elena Institute for Cancer Research, Rome, Italy), were used in the present study: FRM and M14. FRM was recently established from a melanoma patient while M14 is a long established melanoma cell line. Cells were grown in RPMI 1640 medium with 10% (v/v) FBS in humidified incubator with 5% CO_2 _at 37°C and sub-cultured twice a week at 1:3 and 1:5 split ratio for FRM and M14, respectively. For ConA treatment, cells were seeded at 3.0 × 10^4 ^cell/cm^2 ^and allowed to attach overnight. The culture medium was then discarded and replaced with fresh medium containing 10 nM ConA and cells incubated for a further 24 h before the assays.

Phoenix A cells [31] is a producer cell line for the generation of helper free ecotropic retroviruses. Derived from the 293T Human embryonic kidney line, Phonenix A are highly transfectable using either calcium phosphate or lipid-based transfection protocols and allow the production of infectious progeny within a few days. The presence of an IRES-CD8 surface marker expression cassette downstream of the reading frame of the gag-pol construct offers the advantage to monitor the stability of the producer cell population's ability to produce the gag-pol proteins. Most importantly, both gag-pol and env constructs are under different non Moloney promoters thus minimizing the recombination potential with the introduced retroviral construct. Phoenix A cells were grown in High Glucose DMEM medium supplemented with 10% FBS. Cells were never allowed to reach confluency and were passaged twice a week at a 1:4/1:5 split ratio.

### Transfection procedure

Phoenix A cells were harvested by trypsinization and replated at 3,3 × 10^4 ^cell/cm^2 ^in T-75 flasks in complete D-MEM. After 24 h the medium was changed with 13.6 ml of complete D-MEM containing 25 μM Cloroquine diphosphate and the cells were incubated for 30 min at 37°C. At the same time, the DNA Calcium Phosphate co-precipitate mixture was prepared (i.e.: 30 μg of either LZRSpBMNZ or LZRSpBMNZ-E5 plasmid in 0.7 ml 0.25 M CaCl_2_, successively added with 0.7 ml 50 mM N,N-bis (2-hydroxyethyl)- 2-aminoethansulfonic acid). After 30 min at room temperature, the 1.4 ml Calcium Phosphate mixtures were slowly added to the flasks under delicate agitation. After a 12 h incubation at 37°C in a 5% CO_2 _atmosphere, the medium was removed, the cells washed once with PBS, added with fresh complete D-MEM and incubated at 32°C with 5% CO_2 _for 48 h. The medium containing the E5 bearing – or the empty, negative control, -retroviral progenies were removed and centrifuged at 1000 × g for 10 min to pellet cell debris. Clarified supernatant were harvested and either used immediately for infection or aliquoted and stored at -80°C for later use.

### Infection procedure

24 h before infection, melanoma cells were harvested and replated at 2.0 × 10^4 ^cell/cm^2 ^into T-25 flasks. The infection mixtures were prepared by adding 1.5 ml of D-MEM containing either the E5 retrovirus or the empty retrovirus with 1.5 ml of complete D-MEM. Polybrene (5 μg/ml) was then added to each flask directly at the moment of infection. Flasks were then centrifuged at 190 × g for 30 min at room temperature and incubated for 24 h at 32°C in a 5% CO_2 _atmosphere. The medium was then changed with fresh, complete D-MEM and the cells incubated at 37°C with 5% CO_2 _for further 48 h. Surviving cells, roughly 40% of the challenged cells, were then washed twice with PBS and replated at 2 × 10^4 ^cell/cm^2^. The efficiency of infection procedure was measured in a pilot experiment by a dilution limit PCR strategy showing an almost even end point for E5 and the single copy beta-globin reference sequence (data not shown). This finding is compatible with an above 50% infection of target cells carrying 1 to 10 copies of proviral DNAs and is in tune with the results expected on the basis of theoretical considerations. The presence of the proviral E5 DNA and of the E5 specific mRNA was confirmed by PCR and RT-PCR as below described. Cells infected with the control retrovirus were briefly referred to as "control cells" throughout the paper.

### PCR and RT-PCR

Analyses were performed as previously described [27]. Total DNA and RNA were simultaneously extracted from exponentially growing cell cultures by the Tri-Reagent commercial kit (Molecular Research Centre, Cincinnati, OH) used according to the supplier's instruction. The quality of RNAs was evaluated by the A_260_/A_280 _ratio and by visual inspection of ethidium bromide stained formamide agarose gel electrophoresis under UV-B trans-illumination. 1 μg of DNAse digested total RNA and 0.2 μg DNA were amplified in a 50 μl volume of Superscript One-Step (RT)-PCR Platinum TAQ reaction mixture completed with 500 nM up-stream and down-stream primers and 1.5 mM Mg^2+^. For RT-PCR, the reverse transcription was carried out at 45°C for 30 min. Samples were then heated to 95°C for 150 s to inactivate reverse transcriptase and to activate Platinum TAQ Polymerase. Amplification consisted in 35 cycles under the following conditions. For E5: annealing at 94°C for 50 s, extension at 45°C for 50 s and denaturation at 72°C for 60 s and a final cycle with a 10 min long extension. DNA digested non retrotranscribed negative controls were maintained at +4°C during the RT step and placed in the thermal amplifier at the beginning of RT inactivation step (95°C for 150 s). For tyrosinase: annealing at 52°C for 30 s, extension at 73°C for 60 s and denaturation at 95°C for 45 s and a final cycle with a 5 min long extension. For E5 the E5P65 sense (TGC ATC CAC AAC ATT ACT GGC G) and E5M3AS antisense (AAC ACC TAA ACG CAG AGG CTG C) primers were used; for human tyrosinase the primers were Hu-TYR1 (TTG GCA GAT TGT CTG TAG CC) and Hu-TYR2 (AGG CAT TGT GCA TGC TGC TT) as suggested by Calogero et al. [32].

### Cell viability, cell proliferation and cell specific metabolic activity

Cell viability was measured as already described [27], Briefly, cells were seeded in 96-well microplates at a density which allowed an exponential growth rate for the following 5 day incubation (i.e. 1.0 × 10^4^/well for M14 and 1.6 × 10^4^/well for FRM). At 24 h intervals the cells were challenged with 1.25 mg/ml MTT in a 100 μl volume of fresh medium containing 0.1% FBS [33]. After 2 h of incubation the monolayers were then decanted, washed twice with PBS and the reduced insoluble dye eluted by 100 μl of isopropanol/HCl 0.04 N. The cell viability was then assessed through the MTT reducing activity evaluated by the A_540 _– A_750 _difference measured by a microplate reader (Labsystem Multiscan MS – Thermo Fisher Scientific, Inc. Waltham MA).

Cell proliferation was measured by the growth curve as already described [34]. Briefly, cells were seeded in 96-well microplates at the same density as above. At 24 h intervals the monolayers were stained with Crystal Violet (CV), the dye was eluted by means of 33% acetic acid and the cell number in each well was estimated by the A_540 _measured in a microplate reader (Labsystem).

Considering that cell viability assay does actually measure the total reducing activity within a tissue culture, and considering that such a global activity may largely vary according to culture conditions, cell environment and phenotypic status, to gain information about a possible modulation of the metabolic activity within E5 expressing cells, the cell specific metabolic activity was calculated. This is the simple MTT/CV absorbance ratio, expressed in arbitrary units, and gives information about the average metabolic activity of single cells.

For each assay a set of at least four different experiment was considered. Each experiment consisted of eight independent replicas.

### Acridine orange fluorescent staining

To visualize acidic organelles, Acridine orange (AO) was used [35]. AO is a fluorescent probe that emits green at low concentration and orange at high concentration. To determine the effect of treatments on endocellular compartment pH, cell cultures were seeded onto multiwell microscope slides and allowed to attach overnight. The culture medium was then replaced with non supplemented medium or medium containing 10 nM ConA or medium containing the retrovirus. After 24 h, AO (5 μg/ml) was added and incubation continued for another 20 min. The slides were fixed with 2% formaldehyde in PBS and processed for fluorescence microscopy with a Zeiss 466301 microscope. An Olympus Camedia C5060 was used for colour photography.

### Anchorage independent growth assay

A 2 ml of 0.5% agarose gel in RPMI at 10% FCS was poured in each 35 mm well of a plastic plate and allowed to solidify at room temperature for 2 hours in a laminar flow hood. Then a 0.5 ml of a 0.33% agarose gel containing 250 cells was overlaid on top, allowed to stand for 30' at +4°C and subsequently incubated at 37°C. After a 12–16 days incubation the cell growth was evaluated by bright field observation under low magnification and growing colonies photographed.

### Western blot analysis

Immunoblot analysis was performed as previously described [36]. Cell lysis was carried out at 4°C by sonication for 1 min in Media I (0.32 M sucrose, 10 mM Tris-HCl, pH 8.0, 0.1 mM MgCl_2_, 0.1 mM EDTA, 1 mM phenyl-methyl-sulfonyl-fluoride (PMSF) and 10 μg/ml aprotinine) and lysates were stored at -70°C until use. Protein content was determined by the Bio-Rad Protein Assay (Bio-Rad Laboratories Srl, Segrate, Italy). Proteins were separated by 12% SDS-PAGE and transferred to PVDF membranes in 25 mM Tris, 92 mM glycine containing 20% (v/v) methanol at 110 V for 1 h. Following transfer, membranes were placed for 1 h in blocking buffer (bovine serum albumin 3% in T-TBS). For tyrosinase detection, membranes were probed first with 10 ml of blocking buffer containing goat anti-tyrosinase polyclonal antibody (Santa Cruz Biotechnology Inc., CA) (1:500) for 1 h at 27°C, followed by 10 ml of blocking buffer containing horseradish peroxidase-conjugated rabbit anti-goat IgG (1:5000) for 60 min at 27°C. Protein bands were visualized using luminol-based enhanced chemo-luminescence as described by the manufacturer (Perkin-Elmer Life Sciences). Densitometric analysis was performed using Scion Image (PC version of Macintosh-compatible NIH Image).

### Tyrosinase activity assay

Cell monolayers were treated with trypsin/EDTA; suspensions washed with PBS and pellets recovered by centrifugation at 250 × *g *for 10 min. Cells were lysed by sonication (six times for 5 seconds each) in 0.5 ml of 0.1 M Na-phosphate buffer, pH 6.8, containing 0.1 mM PMSF. After centrifugation at 7,000 × g for 10 min, tyrosinase activity was assayed on supernatant according to Iozumi et al. [37]. Fifty μl of sample was incubated in 0.5 ml of a reaction mixture containing 0.1 mM L-tyrosine, 2 μCi per ml of [^3^H] tyrosine, 0.1 mM L-DOPA and 0.1 mM PMSF in sodium phosphate buffer 0.1 M (pH 6.8). After 2 h at 37°C, the reaction was terminated by the addition of 1 ml of charcoal (10% wt/vol in 0.1 N HCl). Samples were centrifuged at 2000 g for 10 min, the supernatant was removed and mixed with scintillation cocktail, and radioactivity was determined using the LS 6500 scintillation system (Beckman, U.S.A.).

### Treatment with cytotoxic agents

Cells were incubated with 30 μM DHBA or BSO in RPMI 1640 medium with 10% (v/v) FBS in humidified incubator with 5% CO_2 _at 37°C. After 48 h incubation cell viability was determined by MTT method, as previously described.

### Statistical analysis

For tissue culture assays a set of at least four different experiment was performed and each data point within any single experiment is the mean (± SD) of eight independent replicas. P values for cell proliferation and cell viability were calculated respect to the corresponding value T = 0. the normal data distribution among samples was assessed by the Shapiro – Wilk test and the Parametric (T Student) or non-Paramentric (Mann-Whitney) test were used accordingly. Standard deviations (SD) were reported for cell specific activity ratios and for the relative tyrosinase expression.

## Results

### The isolated E5 HPV 16 oncogene can be expressed in melanoma cells

**Figure 1:**
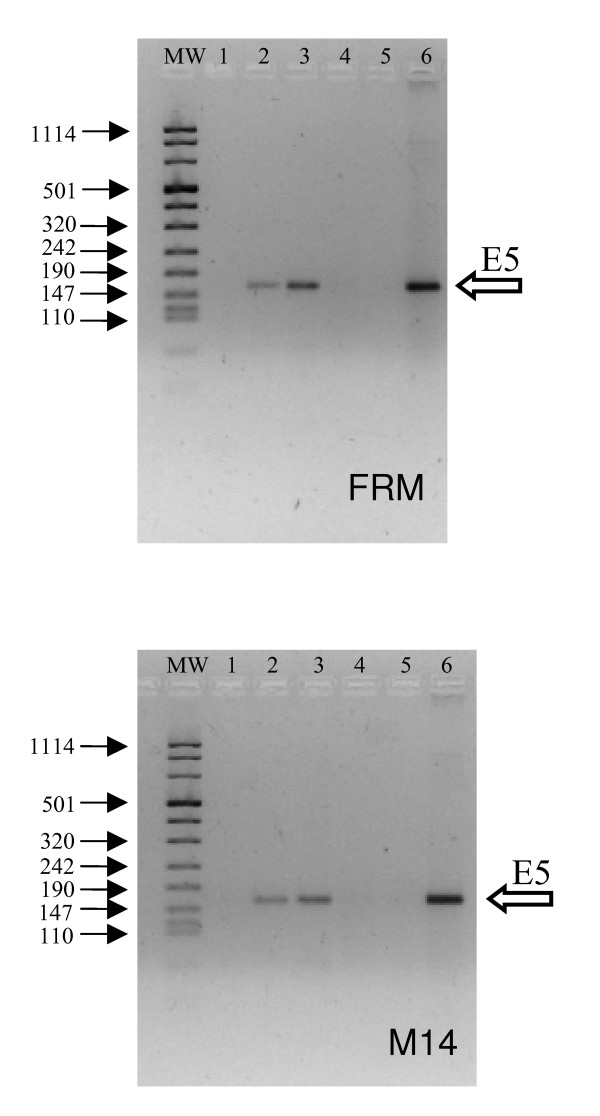
**Presence of HPV-16 E5 DNA and expression of the specific mRNA in M14 and FRM cells after infection with HPV-16 E5 retroviral vector**. The retroviral vector containing HPV-16 E5 gene was obtained by the transfection of Phoenix A retroviral producer cells with the LZRSpBMNZ-E5 plasmid. The control retroviral vector was obtained by the transfection of Phoenix cells with the empty LZRSpBMNZ plasmid. Cells were infected with either recombinant retrovirus or with the control retrovirus. Total DNA or RNA (1 μg) extracted from cells 96 h post infection were reverse transcribed and amplified with E5P65 sense (TGC ATC CAC AAC ATT ACT GGC G) and E5M3AS antisense (AAC ACC TAA ACG CAG AGG CTG C) primers. Upper panel: FRM cells; Lower panel: M14 cells. Lane 1: DNA from cells infected with the control retrovirus; Lane 2: DNA from cells infected with the HPV-16 E5 retrovirus; Lane 3: DNA digested total RNA from cells infected with the HPV-16 E5 retrovirus; Lane 4: Non retrotrascribed DNA digested total RNA from cells infected with the HPV-16 E5 retrovirus; Lane 5: No template negative control; Lane 6 positive control (0.5 μg Siha cell DNA). MW: DNA molecular weight marker VIII (Roche Biochemicals SpA): arrows on the left-hand side indicate the bp length of some reference bands. The band with size of 160 bp (left sided empty arrow) demonstrate the presence of viral E5 sequence and its transcription. Four independent experiments gave similar results.

**Figure 2:**
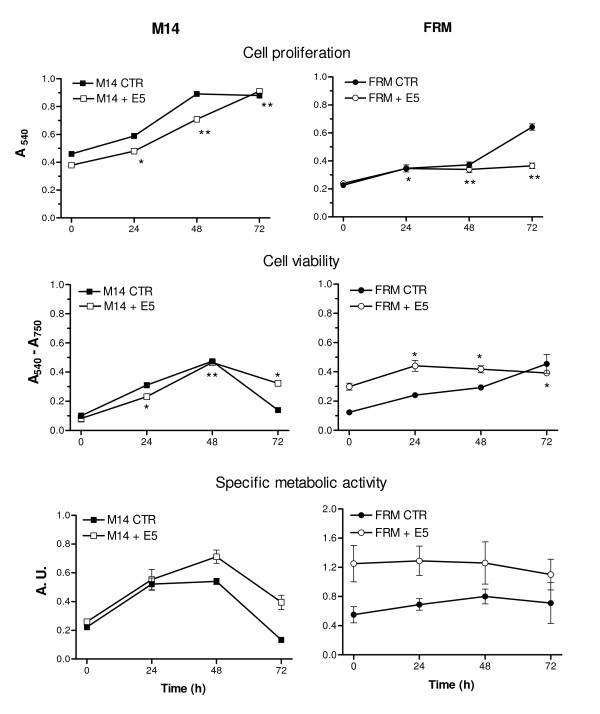
**Effect of HPV-16 E5 expression on the proliferation, cell viability and on cell specific metabolic activity of M14 and FRM melanoma cells**. Cell proliferation (upper row) was slightly decreased in E5 expressing cells (empty symbols) as compared with control cells (full symbols). The cell viability of E5 expressing cells and control cells is shown in the middle row. The cell specific activity of E5 expressing cells (lower row) was higher than that of control cells. This effect, sharply evident in FRM cells appeared slighter in M14 and indicates an increased oxidative metabolism in E5 expressing cells. Values are the mean ± S.D. of eight independent replicas and are derived from a representative experiment in a set of four. Statistical comparison of E5 expressing cells was made using either parametric (Student's *t*-test) or non paramentric (Mann – Whitney test) according to the results of the Shapiro – Wilk assay. (* = *p *< 0.05; ** = *p *< 0.005). The specific metabolic activities are calculated as the simple cell viability/cell proliferation ratio (MTT/CV ratio) and are expressed in arbitrary units as the mean of four different experiments ± SD.

HPV 16 E5 is a small hydrophobic molecule expressed at very low levels in keratinocytes at early stages during viral infection and appearing to be critically linked to viral pathogenic potentials. Two amelanotic melanoma cell lines, FRM and M14, were infected with a HPV 16 E5 expressing retroviral vector and compared with the same lines infected with an "empty" retrovirus. After the infection with the E5 retroviral construct, the presence of cDNA for the E5 oncogene, as well as the corresponding mRNA, was shown by PCR and RT-PCR both in M14 and FRM cells (Fig. 1). Subsequently we investigated whether the E5 oncogene can be tolerated in these cells. Despite the high hydrophobic structure of the E5 protein would suggest a rather toxic effect, the expression of this viral oncogene had almost no effect on cell morphology (data not shown), cell proliferation and cell viability, while a clear increase of the cell specific metabolic activity (more evident in FRM than in M14) was seen in E5 expressing cells (Fig. 2). These characteristics were rather stable being observed in both cell lines as far as the HPV 16 E5 oncogene was retained (at least 4–6 passages *in vitro*). Taken together these data indicate that the isolated HPV 16 E5 oncogene can be expressed in amelanotic melanomas and that its expression, devoid of any immediate gross cell toxicity, induces the fine modulation of selective cell activities.

### E5 expression modulates endosomal pH and restores tyrosinase activity

**Figure 3:**
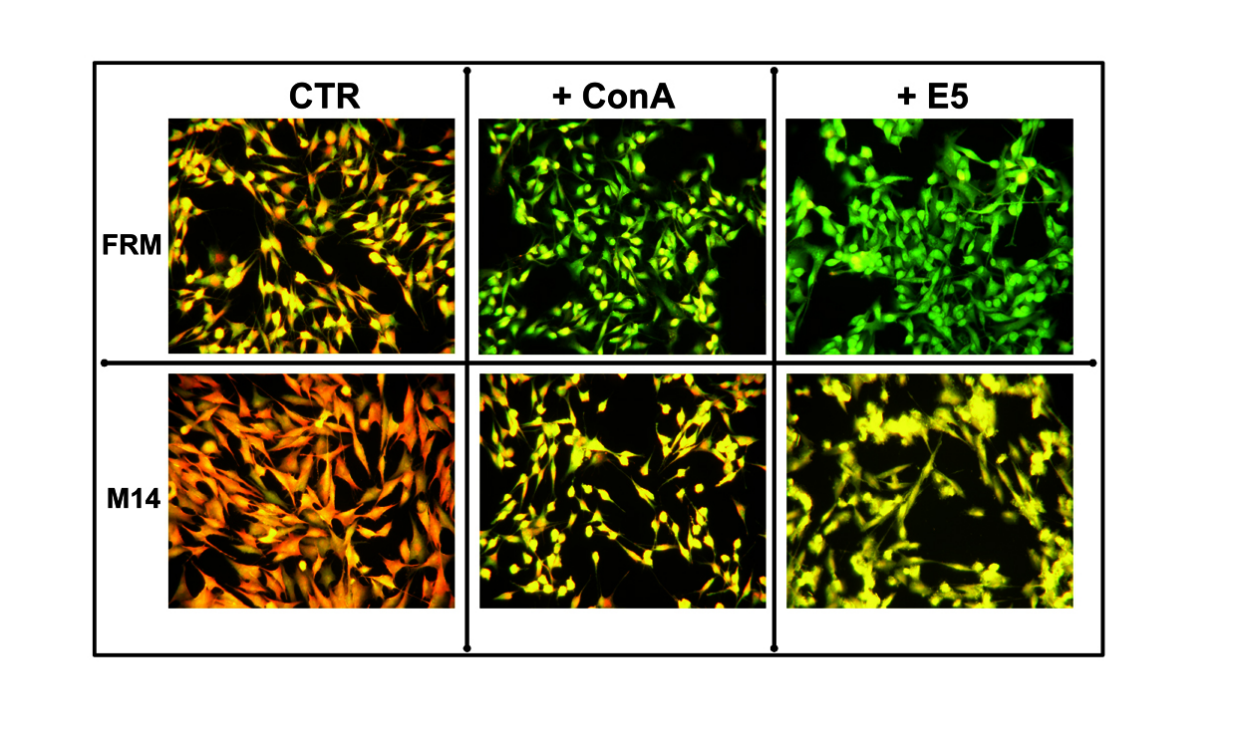
**Effect of HPV-16 E5 expression on intracellular pH in FRM and M14 melanoma cells**. Cells infected with the control retrovirus (CTR), cells treated with 20 nM Con-A (+ ConA) or cells expressing the HPV-16 E5 (+ E5), were stained with AO as described. The loss of orange fluorescence and the appearance of green fluorescence in cells treated with ConA or expressing E5 indicate the alkalinisation of endocellular organelles. A representative experiment in a set of four.

Being well accepted the biochemical interaction of E5 with the V-ATPase proton pump, we investigated if the infection with E5 could determine pH changes in FRM and M14 cells. The fluorescent stain Acridine Orange (AO) used for analysis is an acidotropic weak base which is taken up by living cells and accumulates in acidified compartments such as lysosomes, and melanosomes. When AO accumulates at high concentrations in acidic environment the fluorescence is orange; while at low concentration AO emits green [33]. The effect of E5 expression on endosomal pH is shown in Fig. 3. In E5 expressing cells (+E5), the replacement of orange fluorescence with green fluorescence indicated the raise of intracellular pH with respect to control cells. The addition of the proton pump inhibitor Con-A, a recognised alkalinizing agent, to control cells determined a similar colour change of fluorescence indicating that alkalinisation occurred. In both cases the colour change of fluorescence staining was particularly evident in FRM cells.

**Figure 4:**
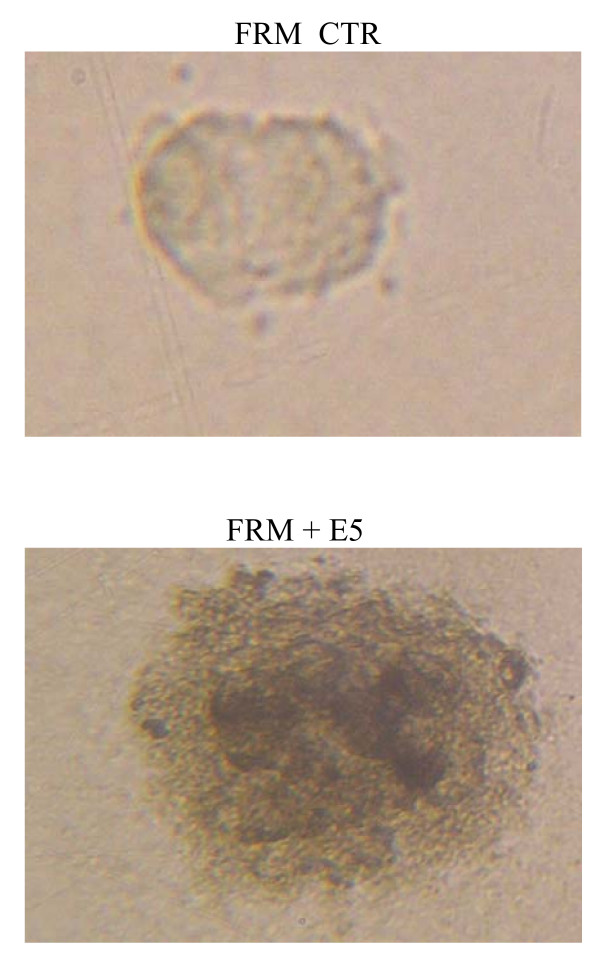
**Effect of HPV-16 E5 expression on tyrosinase activity and pigment deposition and anchorage independent growth of amelanotic melanomas**. Colony formation under anchorage independent culture conditions. The E5 expressing FRM cells displayed a moderated colony formation activity and a variable degree of pigment deposition while no colony nor pigmentation could ever been shown among control parental cells. Similar results were shown with M14 cells (data not shown). A representative experiment in a set of 3.

The alkalinisation of endocellular compartments in the E5 expressing cells was accompanied by the ability to survive in anchorage independent conditions and by a mild deposition of pigment (Fig. 4). These two characteristics are typical of melanomas growing in well oxygenated contexts while totally absent in control cells and in melanomas growing in hypoxic conditions (e.g. during metastatic growth within compact tissues) [38,39]. Thus following E5 expression and pH modulation the whole melanin synthesis pathway was reactivated indicating a partial reversion of the melanomas phenotype.

**Figure 5:**
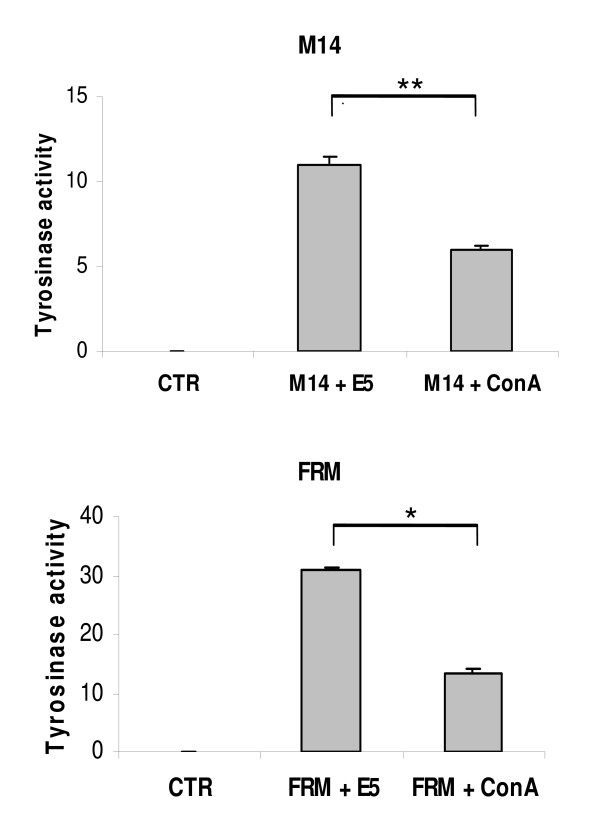
**Tyrosinase activity in FRM and M14 melanoma cells under control conditions, in cells treated with ConA and in HPV-16 E5 expressing cells**. Tyrosinase activity was measured in FRM and M14 melanoma control cells (CTR), in cells treated with ConA (+ ConA) and in HPV-16 E5 expressing cells (+ E5). Cells were lysed by sonication as described in Materials and Methods, Enzymatic activity was assayed by measuring the amount of [^3^H] labelled water produced after incubation for 2 h at 37°C in reaction buffer containing [^3^H] tyrosine. Results are given as nmoles [^3^H]_2_O formed/h/mg protein. The mean ± SD of four independent experiments are depicted. Statistical comparison was made using the non parametric Mann – Whitney test. (*) = *p *< 0.05; (**) = *p *< 0.005. CTR cells did not show enzyme activity. Treatment with V-ATPase inhibitor or E5 expression restored the catalytic activity of the enzyme with the E5 oncogene associated with higher levels of activity.

The tyrosinase activity in E5 expressing or Con A-treated FRM and M14 cells was then determined. As seen in figure 5 the enzyme activity was clearly evident in both E5 cell lines as well as in ConA treated cells, while no activity, as expected was detected in control cells. The rise of enzyme activity was more pronounced in FRM than M14 cells and considerably higher in E5 expressing than in ConA-treated cells.

### The expression of HPV 16 E5 oncogene does not modulate tyrosinase mRNA nor protein levels

**Figure 6:**
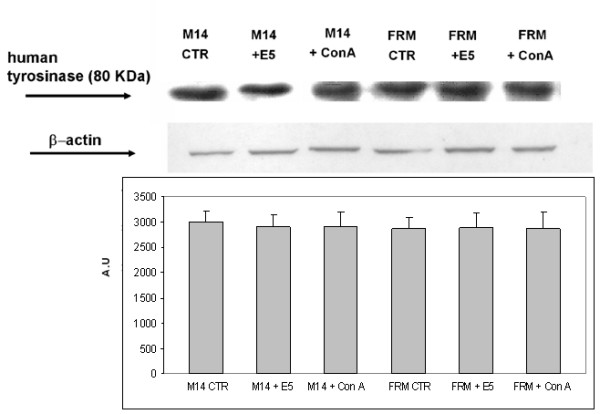
**Expression of HPV-16 E5 oncogene does not affect tyrosinase mRNA transcription and protein expression levels**. Tyrosinase mRNA levels were evaluated by RT-PCR in FRM and M14 melanoma control cells (CTR), in cells treated with 20 nM Con-A (+ ConA) and in cell expressing the HPV-16 E5 (+ E5). Panel a) – Total mRNA (1 μg) was reverse transcribed and amplified with HuTyr-1/HuTyr-2. Four independent experiments gave similar results. All the samples showed similar levels of tyrosinase mRNA. Western blot analysis (panel b) and densitometric quantisation (panel c) of the chemo-luminescent signals of tyrosinase protein levels. No protein modulation was observed under any experimental condition. Results represent the mean ± standard deviation (SD) of four independent experiments. (A.U. = Arbitrary Unit).

In order to understand if the onset of melanotic phenotype and tyrosinase activation following the E5 expression depends on a modulation of tyrosinase transcription and/or protein expression, we determined the tyrosinase mRNA and protein levels by RT-PCR and by Western Blot (WB) analyses, respectively. Figure 6 shows that the expression of E5 oncogene had no effect on tyrosinase mRNA levels both in M14 and FRM cells and confirmed that in these cell lines the amelanotic phenotype is associated with a fair transcription of tyrosinase mRNA [27]. Moreover, WB analysis showed that tyrosinase protein levels were not modulated in E5 expressing cells in comparison with controls. These results, while confirming the poor connection between pigmentation genes expression and the pigmentary status of melanomas, indicate that the amelanotic phenotype of FRM and M14 cells is indeed related to post-translational regulatory process in melanocytes that express normal amounts of tyrosinase protein.

### The tyrosinase reactivation could be exploited as a target for the development of selective chemotherapeutic agents

**Figure 7:**
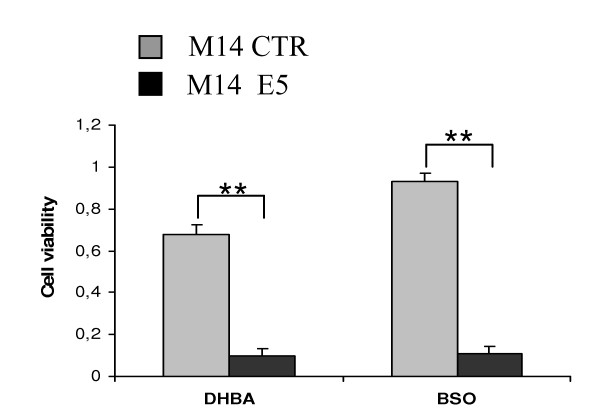
**Effect of HPV-16 E5 expression on the sensitivity of melanoma cells to the tyrosine related antiblastic agents**. M14 control cells (grey bars) or HPV-16 E5 expressing cells (black bars) were incubated with DHBA (up) or BSO (down) at a 30 μM concentration. After 48 h incubation, the cell number was determined using the CV assay as described in the methods section. The E5 expression is associated with a marked sensitivity of melanoma cells to the named anti-tumour agents. Similar results were obtained with FRM cells (data not shown). Reported values are expressed as A_540 _and are the mean ± SD. of eight independent replicas of a representative experiment in a set of four. Statistical comparison was made using the non parametric Mann – Whitney test * *p *< 0.05; ** *p *< 0.005.

Subsequently we wondered whether the above reported endosomal alkalinisation and the reactivation of tyrosinase was associated with modifications in cell phenotype eventually resulting in an altered susceptibility to chemotherapeutic agents. Based on the notion that 3,4-DHBA, a dopamine mimetic pro-drug, is a substrate for tyrosinase with consequent production of toxic intermediates [40] we evaluated its cytotoxic effect in E5 expressing cells. Fig. 7 shows that a 30 μM concentration induced a much stronger impairment of cell viability on E5 expressing melanomas than on the control cells. The same figure shows also that BSO, a well-known inhibitor of glutathione synthesis whose cytotoxic effects are correlated with the level of tyrosinase activity [40], determined a drastic reduction of cell viability in E5 expressing cells, while control cells were scarcely affected.

## Discussion

Pigment deposition takes place in specialized organelles, the melanosomes. In these organelles a number of specific proteins are expressed. Interestingly each of these proteins represents a unique feature of melanocytes and a potential target for the development of selective therapies or elective diagnostic methods for the malignant melanoma [41,42]. Regulation of melanogenesis at transcriptional level is mostly controlled by the microphtalmia transcription factor, however the amelanotic phenotype may also result from post-translational mechanisms in cells expressing normal amounts of pigmentary proteins. This regulatory level has been shown to be important in determining skin and hair colour and pigmentary phenotype of malignant melanomas [37,24].

The fast growing incidence of malignant melanomas in the last decades coupled with the lack of satisfactory treatments for advanced melanomas underline the urgency for a better understanding of their biology and greatly stimulated research in this area. To investigate the possibility to modulate the biological behaviour of amelanotic melanomas through the modulation of the organellar pH, we expressed the HPV 16 E5 oncogene in the FRM and M14 cells and evaluated the implications of such an expression on the cell phenotype. Both are amelanotic cell lines expressing normal levels of tyrosinase maintained in an inactive state by the acidic endosomal pH, as demonstrated by the tyrosinase restoration following the selective inhibition of the V-ATPase by ConA treatment.

The HPV 16 E5 oncogene is a small, highly hydrophobic protein of 83 aminoacids that localizes in endocellular membrane and exhibits only weak transforming activity [6,43]. Within the context of the viral genome it has the function of enhancing the ligand dependent EGF Receptor activation [12] thus resulting in a longer persisting, higher producing viral infection. Once expressed as isolated protein, E5 is mostly found in the endoplasmic reticulum (ER) membranes and at a much lower abundance in the Golgi membranes and endosomes. In ER, through a hydrophobic interaction, the E5 protein would stably associate with 16 kDa subunit of V-ATPase, preventing its assembly into the mature form and therefore suppressing the endosomal acidification [11]. However there is no generalized consent on this mechanism and other authors, based on the failure to induce V-ATPase inhibition in some models [44] and on the report that E5 disrupts actin filaments in fibroblasts [23], proposed that E5-mediated suppression of the endosomal acidification occurs through the disruption of the membrane trafficking responsible for the fusion of early endosomes with the highly acidic mature para-nuclear endosomes[23]. Moreover other E5 indirect mechanisms may be hypothesised based on its complex modulation of cell proteome and membrane lipids and proteins composition [45-47].

Following the infection with a retrovirus construct bearing the HPV-16 E5 sequence, the E5 specific mRNA could be consistently detected in FRM and M14 cells up to thirty days post infection. The E5 viral specific mRNA was expressed at a level comparable with the one of the GAPDH housekeeping reference gene. The E5 expression was well tolerated with almost no cytotoxic effect and no modification of cell morphology. Expectedly, as revealed by experiments with AO, the E5 expression was associated with a relevant modification of the endocellular pH and with a neat re-activation of the tyrosinase enzyme. These data are in favour of the hypothesis that E5 protein does indeed act through an interaction with 16 kDa subunit c of the V_0_-ATPase sub-complex. In fact, in amelanotic melanomas the most of tyrosinase and of other melanogenic proteins, instead of being transported to the Golgi and endosomes for further processing and glicosilation, due to the acidic environment, are retained in the ER where they are rapidly degraded by proteasome [48]. Conversely, the maturation of tyrosinase to the enzymatically active form (figure 4b) indicate the elevation of the endocellular pH to a near neutral value following the V-ATPase complex inhibition thus supporting the hypothesis of an interaction of the E5 with the 16 kDa sub unit c. This interaction could reasonably occurs in the ER where the 16 kDa V-ATPase subunit is synthesized and where most of E5 is localized. However we could not provide a positive evidence for a direct interaction and, considering the multifaceted cellular effects of E5, other indirect mechanisms may be envisioned. Namely the modifications of membrane lipids compositions and functions [45,46] and the deep modifications of cell transcriptome [47], both obtained in HaCaT cells, have the potentials, either alone or in combination, to modulate the proteins and organellar functions without implying any direct physical E5/subunit c interaction.

The E5 expressing cells proved able to sustain the melanin deposition and to survive in anchorage independent culture conditions (figure 4c) thus confirming and extending the observation on mouse embryo fibroblasts [17] and human epithelial HaCaT cells [49] already reported. According to Zhang et al [37], these two features are associated with a reduced growth ability and represent the hallmark of melanomas adapted to grow in well oxygenated tissues. Conversely, a high growth rate, the ability to grow in adherence as in compact lesions and the lack of pigmentary activity (as a consequence of the environment acidification due to the high levels of glycolytic activity -the Warburg effect-), are typical of those melanomas adapted to grow in highly hypoxic condition of fast growing metastases. In this perspective the discussed results are consistent with the hypothesis of a more differentiated phenotype. Indeed following E5 expression and the restoration of a near neutral pH, in addition to the correct maturation of tyrosinase, a global re-organization of the endocellular trafficking occurs. Such a reorganization permits the adequate processing of the many pigmentary proteins through several different pathways and their correct cooperation into the multi-step process of pigment deposition. As a whole these data stand against the hypothesis that the E5 alkalinisation of cellular pH takes place through the subversion of endocellular trafficking, which is on the contrary restored, at least as far as melanogenesis is concerned. Conversely they support the view that the E5 protein, once expressed in an intact human cell, directly or indirectly modulates V-ATPase proton pump with a wide range of orchestrated functional consequences. Finally restoration of the melanogenic phenotype is associated with a clear elevation of cell reducing activity, consistent with a partially re-differentiated phenotype. Once again this result is in line with the hypothesis of a close linkage between the global melanoma phenotype and the cell metabolism which impacts on growth abilities, pathways activation and pigment deposition [36,37].

Being the anaplastic phenotype of melanomas associated with a less favourable clinical outcome and a more severe prognosis [40], we next wondered whether such a reversion could have an impact on response to chemotherapeutic agents. In this work we showed that following the inhibition of V-ATPase by HPV16-E5 the whole melanin synthesis pathway is restored in amelanotic melanoma lines and accordingly these cells appear more responsive to dopamine-mimetic pro-drugs, whose toxicity is related to their oxidation into toxic intermediates i.e. quinones, by tyrosinase-catalyzed reactions. In addition, tyrosinase reactivation is also linked with an increased sensitivity to drugs interacting with other related pathways, as shown by the case of BSO, a GSH depleting drug via the gamma-glutamyl-cysteine synthetase inhibition. Since GSH is a major defence against toxic quinone intermediates through the production of conjugates, GSH depletion results in a severe cell death selectively in those cells where active melanogenesis is present.

In conclusion the expression of the HPV16-E5 oncogene proved able to (partially) revert the malignant phenotype of amelanotic melanomas to a less aggressive, drug responsive state.

## Competing interests

The authors declare that they have no competing interests.

## Authors' contributions

FDD prepared the viral strains and conduced the molecular analysis and helped in coordinating the work. CF participated in data analysis and interpretation and in manuscript preparation. CB and MP have been involved in western blot analysis, enzymatic assays and data interpretation. FP and SM participated in cell culture and cellular work and helped with viral strain preparation. CC participated in study design and critical revision of the manuscript. RC participated in the study design and coordination and helped to revise the manuscript. FDM conceived of the study, participated in its design and coordination, has been involved in data analysis and interpretation and helped to draft the manuscript. All authors read and approved the final manuscript.
